# Trends in Financial Barriers to Medical Care for Women Veterans, 2003–2004 and 2009–2010

**DOI:** 10.5888/pcd10.130071

**Published:** 2013-10-24

**Authors:** Chris Delcher, Yanning Wang, Mildred Maldonado-Molina

**Affiliations:** Author Affiliations: Yanning Wang, Mildred Maldonado-Molina, University of Florida, Gainesville, Florida.

## Abstract

**Introduction:**

Women veterans are a fast-growing segment of the veteran population, yet they face many barriers to medical care. The objective of this study was to examine factors that put women veterans at risk for a financial barrier to medical care.

**Methods:**

We conducted repeated cross-sectional analyses of data from the 2003, 2004, 2009, and 2010 Behavioral Risk Factor Surveillance System. We used weighted logistic regression to examine the risk of a financial barrier to medical care as the primary outcome in a multivariate model controlling for factors in health-related domains.

**Results:**

In 2010, there were an estimated 1,719,750 (11.6%) working-aged veterans who needed to see a doctor in the previous 12 months but could not because of cost. For women, 13.4% faced this financial barrier. Over the study period, facing a financial barrier was consistently associated with insurance coverage, physical and mental distress days, and having children in the home. Other associations emerged in particular years, such as binge drinking in 2010. The trends for women veterans relative to men and for younger women veterans relative to older women veterans show reduction in financial barriers to health care.

**Conclusion:**

The Veteran’s Health Administration (VHA) should continue efforts to reduce financial and other barriers, especially among the higher risk groups we identified. This will help meet the VHA’s objectives of providing comprehensive care to all veterans including women.

## Introduction

Women are a growing proportion of veterans in the United States (1,2). The focus in our study is on women veterans in the general population during the Operation Enduring Freedom, Operation Iraqi Freedom, and Operation New Dawn (OEF/OIF/OND) eras that faced a financial cost barrier to medical care. A large number of all veterans are uninsured or forgo or delay care because of a financial burden (3–6).

For women veterans, functional status and general, physical, and mental health are all substantially worse for those facing a financial barrier to medical care (3). Compared with their civilian counterparts, women veterans are more likely to report poor physical and mental health, divorce, remain divorced, and be homeless (3,5,7). Women veterans and men veterans seek and use health care differently from each other — the women veterans tend to be younger, use more outpatient services, receive mental health diagnoses more frequently, need more mental health service, and have a service-connected disability (1,8). Fifteen percent of women veterans use the Veteran’s Health Administration (VHA) for their health care needs (1). Institutional barriers include lack of benefits knowledge, failure to meet eligibility criteria, limited availability of women’s services, lack of provider training in women’s health needs, and women’s negative perceptions of VHA care (1,5,9). It is important to understand additional barriers, including financial, to medical care in the women veteran population.

The primary objective of our study was to examine sex disparity associated with a self-reported financial barrier to medical care using a nationally representative survey of working-age veterans. We examined sex and age group differences in the prevalence of a financial barrier and other health-related factors. We used a repeated cross-sectional analysis of the Behavioral Risk Factor Surveillance System (BRFSS) survey from the entirety of the OEF/OIF/OND era.

## Methods

We analyzed data from the 2003, 2004, 2009, and 2010 Behavioral Risk Factor Surveillance System (BRFSS), a cross-sectional computer-assisted random-digit–dialed telephone survey conducted by US states and territories with support from the Centers for Disease Control and Prevention (CDC). Although 2011 data are available, that period should be considered a baseline year for data analysis; it is not directly comparable to previous years because of the changes in weighting methods and the addition of the cellular telephone sampling frame (10). The BRFSS is an ongoing data collection program designed to measure behavioral risk factors for the adult population (aged 18 years or older) living in households (11). The median response rate for each year in the analysis was 53.2%, 52.7%, 52.5%, and 54.6%, respectively (12). CDC added the first veteran-related questions to the core questionnaire of the BRFSS in 2003 (13).

Veterans were identified as those respondents indicating military service and not currently on active duty. The BRFSS for 2005 through 2008 did not allow us to distinguish active duty status so we did not include these years in the analysis. We excluded respondents older than 65 because our study focused on working-age veterans. The BRFSS does not capture data from military personnel living in closed quarters or in foreign countries (14). The final sample size for veterans in each year in the analysis was 18,842 (2003), 21,841 (2004), 26,016 (2009), and 25,654 (2010). This study received Health Science Center Institutional Review Board 01 approval from the University of Florida.

We chose 15 variables for analysis: a financial barrier to medical care (outcome), sex (primary covariate), age, race/ethnicity, education level, annual income, marital status, employment status, number of children in household, insurance status, self-reported health status, physical distress days, mental distress days, smoking history, and binge drinking. We chose covariates because they are 1) a priori important for exploring health care access issues in this population and likely confounders and 2) consistently asked in each year in the analysis.

Respondents who answered affirmatively to the question “Was there a time in the past 12 months when you needed to see a doctor but could not because of cost?” were defined as having a financial barrier (outcome). Our primary covariate was sex.

To examine health care coverage status, we used the question “Do you have any kind of health care coverage, including health insurance, prepaid plans such as HMOs [health maintenance organizations], or government plans such as Medicare?” Respondents who answered “no” were considered to be uninsured.

We dichotomized self-reported general health status as “poor and fair” and “good, very good, and excellent.” Two health-related quality of life questions were used to capture physical and mental distress: “How many days during the past 30 days was your physical health not good?” and “How many days during the past 30 days was your mental health not good?” We dichotomized both of these variables to 14 or more days and less than 14 days (15). Smoking status was defined as current smoker, former smoker, or never smoked. Binge drinkers were defined as men reporting 5 or more drinks on 1 occasion or women reporting 4 or more drinks on 1 occasion.

Other sociodemographic characteristics included age (younger group: 18–44 years old, middle-aged group: 45–64 years old, corresponding approximately to service era groups), race/ethnicity (white, black, Hispanic, and other), employment status (employed and unemployed), education level (less than high school graduate, high school graduate, college 1–3 years, and college 4 years or more), marital status (married and unmarried), and children in the household (yes or no). We categorized annual household income as <$15,000, $15,000–$34,999, ≥$35,000, and “unknown.” Proportions of missing income data were higher for 2003 and 2004 than 2009 and 2010. The median annual household income for veterans in 2009 was approximately $35,000 (16).

The BRFSS data are weighted for the characteristics of the sample design — disproportionate sampling by geographic and population density strata, number of telephones and adults in the household, and in terms of respondents’ characteristics (eg, sex, age, race, education) related to key health and risk outcomes on the survey (17). First, we calculated weighted frequencies and the prevalence of cost barriers among women and health-related subgroups of veterans. Second, we performed logistic regression to examine the statistical association (ie, unadjusted odds ratio) of a financial barrier to medical care with all covariates. This step included an age group by sex interaction because previous studies showed that younger (18–44 years) and middle-aged (45–64 years) women veterans differed in VHA care use, insurance coverage, military service experience, and degree of combat exposure (7). Third, we performed multivariate logistic regression to examine the odds of a financial barrier among working-aged veterans, after adjusting for expected confounders and the age group by sex interaction. Results are presented as adjusted odds ratios (AORs) and 95% confidence intervals (CIs). SAS version 9.2 (SAS Institute Inc, Cary, North Carolina) was used to perform analyses.

## Results

In 2010, an estimated 1,719,750 (11.6%) working-aged veterans needed to see a doctor in the previous 12 months but could not because of cost (Table 1). The prevalence of a financial barrier in the veteran population remained stable over the study period. By contrast, the prevalence of a financial barrier increased from 14.3% (2003) to 16.5% (2009) in the general population (data not shown). The prevalence of a financial barrier among veterans was higher for women and for those who were aged 18–44 years, were black or Hispanic, had less than a high school education, earned less than $15,000 a year or were unemployed, were unmarried, had children in the household, were uninsured, were in poorer general health, experienced more physically and mentally unhealthy days, or were a current smoker. Also, in 2010, financial barriers were significantly more prevalent among binge drinkers (14.2% vs 10.9%) than nonbinge drinkers.

**Table 1:** Characteristics of Working-Aged Veterans Who Reported a Financial Cost Barrier to Medical Care in the Prior Year, Behavioral Risk Factor Surveillance System (BRFSS)^a^, 2003–2004 and 2009–2010

Characteristics	2003	2004	2009	2010
**Population estimate, N (%)**	1,793,987 (11.1)	1,747,254 (11.0)	1,747,254 (11.0)	1,719,750 (11.6)
**Survey, N**	1,965	2,406	2,680	2,653
**Sex**				
Female	17.7 (14.4–21.0)	14.1 (11.7–16.6)	15.6 (12.7–18.5)	13.4 (11.3–15.5)
Male	10.6 (9.7–11.5)	10.7 (9.9–11.5)	10.5 (9.7–11.4)	11.3 (10.5–12.2)
**Age group, y**				
18–44	16.4 (14.5–18.4)	15.6 (14.0–17.2)	12.9 (11.2–14.6)	13.5 (11.8–15.3)
45–65	8.4 (7.6–9.3)	8.6 (7.8–9.5)	10.0 (9.2–10.8)	10.3 (9.6–11.1)
**Race/ethnicity**				
White	10.2 (9.3–11.0)	9.8 (9.1–10.6)	9.4 (8.7–10.1)	10.6 (9.7–11.5)
Black	12.7 (9.4–16.0)	13.1 (10.6–15.7)	14.4 (11.9–17.0)	12.5 (10.2–14.7)
Hispanic	16.3 (11.3–21.4)	14.6 (10.1–19.1)	18.1 (12.7–23.5)	14.5 (10.6–18.4)
Other	14.1 (10.1–18.1)	18.9 (14.2–23.6)	14.6 (10.8–18.3)	16.2 (12.2–20.1)
**Education level**				
Less than high school graduate	21.5 (15.6–27.4)	24.6 (18.6–30.5)	24.2 (17.3–31.1)	24.8 (17.8–31.8)
High school graduate	13.0 (11.3–14.8)	13.9 (12.4–15.5)	14.4 (12.7–16.0)	15.0 (13.1–17.0)
College, 1–3 y	12.2 (10.7–13.7)	11.8 (10.4–13.2)	12.7 (11.0–14.4)	12.8 (11.5–14.0)
College, 4 y or more	6.4 (5.2–7.5)	6.1 (5.1–7.0)	5.8 (4.9–6.7)	6.8 (5.8–7.8)
**Annual income, $**				
<15,000	34.6 (29.2–40.0)	34.4 (29.1–39.8)	34.1 (29.6–38.7)	31.8 (27.3–36.3)
15,000–34,999	24.3 (21.0–27.5)	27.1 (23.9–30.3)	25.0 (22.6–27.5)	24.8 (22.2–27.3)
≥35,000	15.1 (12.2–17.9)	17.0 (13.9–20.1)	5.8 (5.0–6.7)	5.7 (5.1–6.3)
Unknown	6.6 (5.7–7.5)	6.1 (5.4–6.7)	11.1 (8.0–14.1)	14.6 (11.1–18.1)
**Marital status**				
Unmarried	17.4 (15.5–19.3)	16.2 (14.6–17.8)	18.6 (16.6–20.7)	20.1 (18.1–22.2)
Married	8.4 (7.4–9.3)	8.8 (7.9–9.7)	8.1 (7.4–8.9)	8.4 (7.6–9.2)
**Employment status**				
Unemployed	15.8 (14.0–17.7)	16.5 (14.8–18.3)	16.7 (15.0–18.3)	18.5 (16.7–20.2)
Employed	9.3 (8.3–10.2)	9.0 (8.2–9.8)	8.6 (7.7–9.6)	8.2 (7.4–9.0)
**Have children in household**				
Yes	13.9 (12.2–15.6)	12.8 (11.4–14.3)	12.9 (11.3–14.5)	12.0 (10.6–13.5)
No	9.5 (8.6–10.4)	10.0 (9.1–10.9)	9.9 (9.1–10.8)	11.2 (10.3–12.1)
**Health care coverage**				
Insured	7.4 (6.6–8.2)	7.3 (6.6–8.0)	7.3 (6.6–8.0)	7.6 (6.9–8.2)
Uninsured	36.0 (32.3–39.6)	35.8 (32.5–39.1)	39.0 (35.0–43.0)	41.3 (37.6–45.1)
**Health status^b^**				
Good to Excellent	8.8 (8.0–9.7)	9.3 (8.6–10.1)	8.9 (8.0–9.8)	8.8 (8.0–9.6)
Fair to Poor	24.2 (21.2–27.2)	20.5 (17.9–23.0)	24.1 (21.7–26.6)	26.7 (24.2–29.3)
**Physical distress^c^**				
≥14 Days	26.2 (22.6–29.8)	22.8 (19.7–25.8)	23.1 (20.3–25.9)	25.2 (21.9–28.4)
<14 Days	9.1 (8.2–9.9)	9.4 (8.7–10.2)	9.6 (8.7–10.4)	9.6 (8.8–10.3)
**Mental distress^d^**				
≥14 Days	27.1 (23.5–30.8)	29.2 (25.7–32.7)	27.7 (24.2–31.1)	30.2 (26.8–33.7)
<14 Days	9.3 (8.5–10.2)	9.0 (8.2–9.7)	9.0 (8.2–9.8)	9.0 (8.3–9.8)
**Smoking status**				
Current	17.7 (15.8–19.6)	18.1 (16.3–20.0)	19.4 (17.2–21.7)	20.2 (18.1–22.2)
Former	7.3 (6.2–8.4)	8.7 (7.6–9.8)	8.9 (7.8–10.0)	9.7 (8.6–10.8)
Never	9.1 (7.7–10.5)	7.9 (6.8–9.0)	8.2 (7.0–9.4)	8.3 (7.1–9.5)
**Binge drinking^e^**				
Yes	12.3 (10.3–14.3)	11.6 (9.8–13.4)	11.1 (9.1–13.0)	14.2 (11.7–16.8)
No	10.8 (9.8–11.7)	10.9 (10.0–11.7)	11.2 (10.2–12.1)	10.9 (10.1–11.7)

Abbreviation: CI, confidence interval.

a Data presented are % (95% CI) unless otherwise indicated.

b All health status questions are self-reported.

c Self-reported days with not good physical health.

d Self-reported days with not good mental health.

e Defined as having 5 or more drinks on 1 occasion for men and 4 or more drinks on 1 occasion for women.

Three primary comparisons are of interest for women veterans: 1) women veterans versus men veterans, 2) women veterans versus the general women population, and 3) younger women veterans versus older women veterans. In 2010, 13.4% of women veterans (n = 227,187) reported a financial barrier compared with 11.3% of male veterans (n = 1,492,563). For this year, the overall sex difference was not significant but 1) middle-aged women veterans were more likely to report a financial barrier (OR, 1.5; 95% CI, 1.1–2.0) than middle-aged male veterans and 2) women veterans reported financial barriers more frequently than their male counterparts in all prior years. Women veterans were less likely to report a financial barrier than women in the general population (Figure). When analyzed by younger and middle-aged age groups of women in both populations, these results were consistent. For 3 of the 4 years, younger women veterans were more likely to face a financial barrier than middle-aged women veterans.

**Figure:**
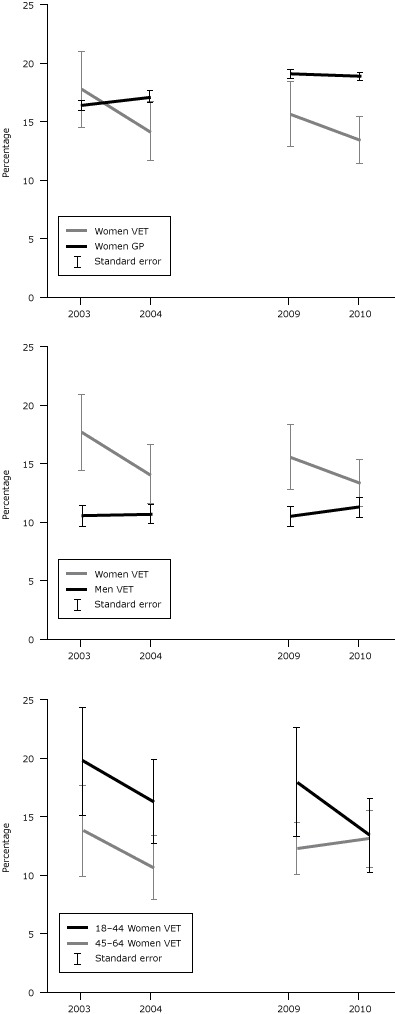
The prevalence (weighted) of reporting a financial barrier to medical care by sex, age, and veteran status, Behavioral Risk Factor Surveillance System (BRFSS), 2003–2004 and 2009–2010. BRFSS data for 2005 through 2008 does not distinguish veteran duty status. Abbreviations: SE, standard error; GP, general population; VET, veteran. **Table:** 

Characteristic	Percentage Reporting a Financial Barrier			
	2003, % (SE)	2004, % (SE)	2009, % (SE)	2010, % (SE)
**Women GP**	16.40 (0.42)	17.11 (0.40)	19.01 (0.40)	18.84 (0.37)
**Women VET**	17.69 (3.30)	14.14 (2.45)	15.60 (2.89)	13.37 (2.09)
**Men VET**	10.57 (0.89)	10.73 (0.82)	10.54 (0.86)	11.33 (0.87)
**18–44 Women VET**	19.77 (4.60)	16.35 (3.59)	17.99 (4.66)	13.49 (3.23)
**45–64 Women VET**	13.89 (3.92)	10.73 (2.75)	12.38 (2.32)	13.22 (2.43)

For brevity, we do not present the bivariate analysis. Table 2 shows the AORs with 95% CIs for reporting a financial barrier in each year of the analysis. For consistency, we report a series of age group by sex linear contrasts (Table 2). The results indicate that age and sex do not interact to modify the likelihood of a financial barrier. After adjustment, race/ethnicity does not appear to be an independent risk factor. In 2010, being unmarried, having children in the household, having poor to fair health, being a current smoker, and a binge drinking were all significant risk factors. The independent effect of health care coverage showed that uninsured veterans were 4 or more times as likely to report a financial barrier as insured veterans in each year. The odds ratio in 2010 (OR, 5.8; 95% CI, 4.7–7.0) was the highest for any year in the analysis. The effect of physical and mental distress days remained significant risk factors in each year; the relative effect of mental health distress appears to be larger than physical distress on financial barriers in more recent years.

**Table 2:** Adjusted Odds Ratios (AORs) for Reporting a Medical Cost Barrier in the Prior Year Among Working-Aged Veterans, Behavioral Risk Factor Surveillance System (BRFSS), 2003, 2004, 2009, and 2010

Variable	2003, AOR (95% CI)	2004, AOR (95% CI)	2009, AOR (95% CI)	2010, AOR, (95% CI)
Sex*Age Contrasts				
18–44 Female*18–44 Male	1.4 (1.0–2.1)	1.1 (0.8–1.5)	1.8 (1.3–2.6)	1.1 (0.7–1.6)
18–44 Female*45–64 Female	1.6 (1.0–2.5)	1.7 (1.1–2.8)	1.5 (1.0–2.2)	0.9 (0.6–1.4)
18–44 Male*45–64 Male	1.7 (1.3–2.2)	1.9 (1.5–2.4)	1.0 (0.8–1.3)	1.3 (1.0–1.6)
45–64 Female*45–64 Male	1.6 (1.1–2.3)	1.2 (0.8–1.7)	1.2 (0.9–1.6)	1.5 (1.1–2.0)
18–44 Female*45–64 Male	2.5 (1.7–3.6)	2.0 (1.4–2.8)	1.8 (1.3–2.7)	1.4 (0.9–2.0)
45–64 Female*18–44 Male	0.9 (0.6–1.4)	0.6 (0.4–0.9)	1.2 (0.9–1.7)	1.2 (0.8–1.6)
**Race/ethnicity**				
White	1 [Reference]	1 [Reference]	1 [Reference]	1 [Reference]
Black	0.9 (0.6–1.3)	1.0 (0.7–1.2)	1.2 (0.9–1.6)	0.9 (0.7–1.2)
Hispanic	1.2 (0.8–2.0)	1.4 (0.9–2.0)	1.6 (1.1–2.4)	1.0 (0.7–1.4)
Other	1.1 (0.8–1.7)	1.6 (1.1–2.2)	1.3 (0.9–1.8)	1.1 (0.8–1.5)
**Education level**				
Less than high school graduate	1 [Reference]	1 [Reference]	1 [Reference]	1 [Reference]
High school graduate	0.9 (0.6–1.4)	0.7 (0.5–1.1)	0.8 (0.5–1.2)	1.0 (0.6–1.5)
College, 1–3 y	1.1 (0.7–1.7)	0.7 (0.5–1.1)	0.8 (0.5–1.4)	1.1 (0.7–1.7)
College, 4 y or more	0.9 (0.5–1.3)	0.6 (0.4–0.9)	0.6 (0.4–1.0)	0.9 (0.6–1.5)
**Annual income, $**				
<15,000	2.7 (1.9–4.0)	3.6 (2.4–5.3)	3.0 (2.1–4.1)	2.2 (1.6–3.0)
15,000–34,999	2.0 (1.5–2.6)	2.5 (2.0–3.1)	2.6 (2.0–3.3)	2.5 (2.0–3.0)
≥35,000	1 [Reference]	1 [Reference]	1 [Reference]	1 [Reference]
Unknown	1.3 (0.9–2.0)	1.4 (1.0–1.9)	1.2 (0.8–1.8)	1.9 (1.4–2.5)
**Unmarried**	1.2 (0.9–1.5)	1.0 (0.8–1.2)	1.3 (1.0–1.6)	1.4 (1.2–1.7)
**Unemployed**	0.8 (0.7–1.1)	1.0 (0.8–1.3)	0.9 (0.7–1.1)	1.1 (0.9–1.4)
**Have children in household**	1.4 (1.1–1.8)	1.1 (0.9–1.4)	1.6 (1.3–2.0)	1.4 (1.2–1.8)
**Uninsured**	4.8 (3.8–6.0)	4.5 (3.7–5.5)	5.4 (4.3–6.7)	5.8 (4.7–7.0)
**Health status^a^**				
Poor to fair	2.1 (1.6–2.8)	1.2 (0.9–1.5)	1.8 (1.3–2.3)	2.0 (1.6–2.5)
Good to excellent	1 [Reference]	1 [Reference]	1 [Reference]	1 [Reference]
**Physical distress days^b^**	2.0 (1.5–2.7)	1.7 (1.2–2.3)	1.4 (1.1–1.9)	1.3 (1.0–1.7)
**Metal distress days^c^**	1.5 (1.1–2.0)	2.3 (1.8–3.0)	2.0 (1.6–2.6)	2.3 (1.8–2.8)
**Smoking status**				
Current smoker	1.4 (1.1–1.8)	1.6 (1.3–2.0)	1.5 (1.2–1.9)	1.7 (1.4–2.1)
Former smoker	0.9 (0.7–1.1)	1.2 (1.0–1.5)	1.2 (0.9–1.5)	1.3 (1.1–1.6)
Never smoked	1 [Reference]	1 [Reference]	1 [Reference]	1 [Reference]
**Binge drinking^d^**	1.0 (0.8–1.3)	0.7 (0.6–1.0)	0.9 (0.7–1.1)	1.3 (1.0–1.6)

Abbreviation: CI, confidence interval.

a All health status questions are self-reported.

b Self-reported ≥14 days with not good physical health.

c Self-reported ≥14 days with not good mental health.

d Defined as having 5 or more drinks on 1 occasion for men and 4 or more drinks on 1 occasion for women.

## Discussion

We found evidence from the general US population that indicates an improvement in accessing health care for women veterans during the OEF/OIF/OND era. In 2010, the prevalence of a financial barrier for women veterans declined to levels similar to those historically reported by men veterans. Women veterans also show increasing improvement relative to nonveteran women in the general population. Although younger women veterans tended to seek medical care less frequently than middle-aged women veterans in the OEF/OIF/OND era, this gap decreased to parity in 2010.

We found that, perhaps for the first time in the OEF/OIF/OND era, reporting a financial barrier was equally as prevalent among younger women veterans as among middle-aged women veterans. This trend coincides with expanded care provisions of the National Defense Authorization Act of 2008, which gave many OEF/OIF/OND veterans eligibility for free VA care for 5 years after leaving the service (18). Furthermore, we did not observe the same trend for younger women in the general population (data not shown). However, it is difficult to interpret this result given the variability in this analysis because of the sample size and the cross-sectional nature of the BRFSS. Future investigations might examine the variability in health care access for veterans as it relates to policy changes, economic conditions or income. A report from the Veterans Administration Office of Policy and Planning indicates that median income fluctuates more dramatically for women veterans than either men veterans or women nonveterans (16).

Experiencing a financial barrier as a function of insurance coverage, physical and mental distress days, children in the home, and binge drinking are worth noting. Not surprisingly, health care coverage had the strongest association with reporting a financial barrier of any independent variable in our study. Uninsured, working-aged veterans were nearly 4 to 5 times more likely to report a financial barrier to medical care than those with insurance. In 2010, we estimate that there were approximately 1.9 million working-aged veterans, approximately 176,000 (9.2%) of whom were women, without health care coverage. This proportion of women among the uninsured is only slightly higher than that reported from the BRFSS in 2000 (8.7%) (6). Also, our analysis suggests that veterans with physical and mental health problems continue to face financial barriers to medical care. This corroborates research conducted by using VA databases (19).

Our study has several limitations. The sample size for women veterans in the BRFSS is stable but still small, leading to high year-to-year variability. Our dependent variable, which is essentially a self-assessment of medical need, is self-reported and subject to bias. For example, women soldiers on active duty underreport health problems perhaps to maintain an appearance of strength among their male peers (14). This type of response bias could persist after leaving active duty. Although we cannot determine the severity of this self-reported medical need for women veterans, research with male veterans suggests that they will forego medical care even for serious symptoms (20). Our results may overestimate group differences if those who reported a financial cost barrier to medical care could not afford care for a short period of time but sought it shortly thereafter. Given that we were interested in examining trends in this population, we chose independent variables that were consistently asked in each year of the analysis. This means that we were unable to include some variables (eg, urban/rural residence, type of insurance plan) that are important but only asked sporadically in the BRFSS.

The strengths of our study include 1) our examination of longitudinal trends from a nationally representative sample of women veterans where several similar studies only use a single year of data, 2) a robust sample size for women veterans in each year, 3) multivariate adjustment for veterans’ sociodemographic characteristics, health care access and use, and health-related characteristics, and 4) comparisons of the prevalence of reporting a financial cost barrier to medical care by sex, age group, and veteran status.

More than 10% of women veterans in the general population during the OEF/OIF/OND era still report a lack of some medical care due to financial barriers. This work adds to ongoing women veterans’ health research by using a nationally representative sample of veterans from the general population. The VHA should continue making efforts to reduce financial and other barriers, especially among some of the higher risk groups identified in this analysis, as it seeks to meet the objectives of providing comprehensive care that “addresses all medical, behavioral, psychosocial, and functional status issues” (p. 1) such as that described in its Patient Centered Medical Home concept (21).
