# Addendum: Vol. 10, October 24 release

**DOI:** 10.5888/pcd10.130071e

**Published:** 2013-11-21

In the article “Trends in Financial Barriers to Medical Care for Women Veterans, 2003–2004 and 2009–2010,” a sentence in the Acknowledgments was omitted. The additional sentence should read, “The authors thank Dr Diane C. Cowper Ripley for her review and Nichole Snyder for manuscript assistance.”

The correction was made to our website on November 6, 2013, and appears online at http://www.cdc.gov/pcd/issues/2013/13_0071.htm.

